# Correlation of fecal calprotectin levels with the detection of treatable enteric pathogens in children with severe acute diarrheal disease in Botswana

**DOI:** 10.1371/journal.pone.0328764

**Published:** 2025-08-21

**Authors:** Muhammad Rehan, Elspeth MacBain, M. Sharif Shajib, Margaret Mokomane, Kwana Lechiile, Tonya Arscott-Mills, Andrew P. Steenhoff, Loeto Mazhani, Cheryl Main, Marek Smieja, Waliul I. Khan, David M. Goldfarb, Jeffrey M. Pernica

**Affiliations:** 1 Department of Pathology and Laboratory Medicine, The University of British Columbia, Vancouver, British Columbia, Canada; 2 Department of Pediatrics, The University of British Columbia, Vancouver, British Columbia, Canada; 3 Department of Pathology and Molecular Medicine, McMaster University Faculty of Health Sciences, Hamilton, Ontario, Canada; 4 Faculty of Health Sciences, School of Allied Health Professions, University of Botswana, Gaborone, Botswana; 5 Botswana-UPenn Partnership, Gaborone, Botswana; 6 Department of Pediatrics, University of Pennsylvania, Philadelphia, Pennsylvania, United States of America; 7 Department of Pediatric and Adolescent Health, University of Botswana, Gaborone, Botswana; 8 Division of Infectious Disease & Global Health Center, Children’s Hospital of Philadelphia, Philadelphia, Pennsylvania, United States of America; 9 Department of Health Research Methods, Evidence, and Impact, McMaster University, Hamilton, Ontario, Canada; 10 Department of Pediatrics, McMaster University Faculty of Health Sciences, Hamilton, Ontario, Canada; 11 McMaster Children’s Hospital, Hamilton, Ontario, Canada; Universidade dos Açores Departamento de Biologia: Universidade dos Acores Departamento de Biologia, PORTUGAL

## Abstract

Diarrheal disease is a leading cause of death among young children globally. Current guidelines recommend supportive treatment of acute diarrhea and using antimicrobials only with presence of blood in the stool. Select enteric pathogens, including *Shigella*, commonly cause disease in high-burden settings; targeted treatment of these pathogens could decrease morbidity and mortality. In settings with limited access to microbiological testing, practical diagnostics are needed to differentiate treatable causes of pediatric diarrhea. Evolving evidence suggests fecal calprotectin (fCal) could help differentiate viral and bacterial gastroenteritis. This study describes a post hoc analysis of stool samples prospectively collected from children hospitalized with severe acute diarrheal disease in Botswana. Specimens were characterized using multiplex PCR panels for selected enteropathogens and assayed for fCal. Stool samples from 312 participants were tested. Samples positive for *Shigella* had significantly higher fCal than samples positive for rotavirus. Stools that were negative for all assayed pathogens had higher fCal values than expected using standard normative values for healthy children in higher-income settings. Given the prevalence of *Shigella* and rotavirus infections in young children globally, fCal may be a useful aid to identify children with acute diarrhea for whom antimicrobials could provide benefit and potentially reduce growth failure and mortality.

## Introduction

Diarrheal disease is a leading cause of mortality in young children living in low- and middle- income countries (LMICs) [1]. It also causes significant morbidity through malnutrition, neurocognitive maldevelopment, and ultimately decreased adult human capital [2]. Certain enteropathogens, such as *Shigella* spp., are likely to have an outsized impact on linear growth and long-term sequelae compared to viral pathogens [3,4]. World Health Organization (WHO) guidelines recommend supportive treatment of acute gastroenteritis (AGE) with addition of antimicrobials only for dysentery or presumed cholera [5]. However, visible blood in stool only occurs in a minority of infections with *Shigella* and other treatable enteric pathogens associated with growth failure and increased mortality (e.g., *Cryptosporidium*, enterotoxigenic *E. coli* [ETEC]) [6–8]. A recent subgroup analysis of a large randomized controlled trial found significant growth and mortality benefit associated with azithromycin treatment of children with non-bloody shigellosis [9], emphasizing that effort should also be put into optimizing the diagnosis and treatment of non-bloody gastroenteritis. However, it is challenging to identify treatable AGE in high-burden settings. Standard stool culture and microscopy require laboratory resources, both in the form of skilled technologists and reagent consumables, that are costly and generally difficult to source and retain in low-resource settings. Culture-based testing is insensitive, so can miss clinically relevant infections and takes days to return. Rapid antigen (immunochromatographic) testing is available and reasonably sensitive for rotavirus [10] but insensitive for norovirus [11] and adenovirus detection [12]. Molecular diagnostics are much more sensitive for pathogen detection and faster than culture but are often not available in resource-limited settings due to high cost (both of device and consumables) and complexity. Emerging Shigella immunochromatographic flow assays and isothermal amplification tests may represent lower-cost solutions but are not yet widely available [13].

Calprotectin is a calcium-binding cytosolic protein in neutrophilic granulocytes and the concentration in feces reflects neutrophil influx into the gut lumen. Fecal calprotectin (fCal) is established as a marker of intestinal inflammation in inflammatory bowel disease [14]. Evolving evidence suggests fCal could discriminate between bacterial and viral AGE, with concentrations of fCal observed to be significantly higher in patients with bacterial gastroenteritis [6,15–20]. Operationalizing point-of-care fCal testing in less-resourced settings may be a practical means of identifying children with AGE who might benefit from antibiotic treatment, as it is simpler, faster and less expensive than traditional culture-based methods or multiplex molecular assays. The objective of this study was to investigate the distribution of fCal in a large cohort of children hospitalized for severe AGE in Botswana and correlate with enteropathogens detected in stool.

## Materials and methods

### Study population and recruitment

This study was a post-hoc analysis of specimens obtained as part of two previous prospective studies; full details of participant recruitment, inclusion and exclusion criteria and informed written consent have been previously described [8,21]. The first prospective study sites were Princess Marina Hospital, Gaborone, Botswana, from 1 May 2011–30 April 2013 and Nyangabgwe Referral Hospital, Francistown, Botswana, from 1 February 2011–31 July 2011 [8]. All inpatients < 13 years of age with acute diarrhea at admission were eligible. Children who developed diarrhea >48 hours after admission to hospital or were discharged within 7 days of diarrhea onset were excluded. Those with additional infective diagnoses at admission (e.g., bacterial sepsis, tuberculosis) were not excluded. The second study sites included Princess Marina Hospital from 1 March 2014–31 Dec 2014, Bamalete Lutheran Hospital (Ramotswa), and Scottish Livingstone Hospital (Molepolole) from 1 Sept 2014–31 Dec 2014 [21]. All children aged 2–60 months hospitalized due to acute non-bloody diarrheal disease were eligible for inclusion.

### Research ethics

The original study protocols covering tissue and data collection were approved by the ethics committees of the Botswana Ministry of Health (Health Research and Development Committee), Princess Marina Hospital, Scottish Livingstone Hospital, University of Pennsylvania, and McMaster University (Hamilton Integrated Research Ethics Board). Informed written consent from all child participant parents/guardians was obtained prior to enrolment. Ethical approval to publish the current data and to include the non-identifiable dataset in a public repository was provided by the Hamilton Integrated Research Ethics Board.

### Laboratory testing

Flocked Fecalswabs™ (Copan Italia, Brescia, Italy) were used to obtain rectal specimens at the time of participant recruitment and were collected, transported, and processed as described previously [22]. For participants drawn from the first study, detection of 15 pathogens (3 viruses, 3 parasites and 9 bacteria) was performed using the Gastrointestinal Pathogen Panel (GPP) assay (Luminex Molecular Diagnostics) on the MAGPIX™ system. The limits of detection for each target in this multiplex assay have been reported [23]. For participants drawn from the second study, two previously validated multiplex PCR assays [22] were performed using the ABI 7500 FAST (Life Technologies, Carlsbad, CA) to detect the following gastrointestinal pathogens: *Shigella/*Enteroinvasive *E. coli* (EIEC), *Salmonella spp.*, *Campylobacter jejuni/coli*, enterotoxigenic *E. coli* LT/ST, *Giardia* and *Cryptosporidium*. These assays were developed as previously described [22,24]. Prior to implementation of the *Cryptosporidium* PCR assay, a commercial antigen detection test (ImmunoCard STAT^®^, Meridian Bioscience) was utilized on bulk stool samples according to manufacturer instructions. All enteric specimens underwent high sensitivity testing for major bacterial enteropathogens but only specimens from the first study underwent testing for viral pathogens.

Stool samples were not sampled randomly for fCal testing. A large number of rotavirus-positive and *Shigella*-positive stools were selected, given the importance of these pathogens, as well as the fact that these pathogens, when isolated, are most likely to be causative of disease [25]. Stools positive for other important pathogens and stools negative for all assayed pathogens were also selected.

Stool samples were frozen and stored at -80C within 24 hours of collection. To extract fCal, Smart Prep Extraction device from Roche Diagnostics was used according to the manufacturer’s instructions. Following homogenization, the extracts were centrifuged at 3000 × g for 5 minutes; thereafter, supernatants were collected and stored at −20°C until analysis. FCal was measured using the ELISA (PhiCal Calprotectin-EIA; Immunodiagnostik) kits according to the manufacturer’s instructions.

### Statistical analysis

Demographic and clinical characteristics were reported using descriptive statistics; medians and quartiles were reported for continuous data and counts and percentages for categorical data. FCal distributions were compared using one-way ANOVA using the Bonferroni post-hoc test to identify significant pairwise differences. A p-value of 0.05 or smaller was considered statistically significant. Analysis was done using STATA IC v16.1 (College Station, TX).

## Results

A total of 312 children had stool samples assayed for enteropathogens and fCal. This included 252/671 from the first study [8] and 60/76 from the second [21]. The total specimens positive for each target and corresponding fCal are listed in Table 1. Co-detection of multiple pathogens was common. Table 1 provides fCal values for all stools positive for a given enteropathogen as well as fCal values for stools positive for only each specific enteropathogen. The supporting information Table S1 provides this information stratified by each original prospective study.

**Table 1:** Enteropathogen detection and fCal levels.

Pathogen	# stools positive	# stools negative	Fecal calprotectin (μg/g)
*Shigella* alone	34 (10.9%) 20	278	338 (126-717) 338 (105-1095)
*Campylobacter* alone	28 (9.0%) 7	284	264 (83-542) 253 (38-680)
ETEC alone	13 (4.2%) 2	294	303 (198-992) 648 (303-992)
*Cryptosporidium* alone	29 (9.9%) 18	264	221 (52-612) 181 (27-594)
*Salmonella* alone	7 (2.2%) 5	305	108 (44-143) 108 (44-125)
Rotavirus alone	100 (39.7%) 97	152	102 (39-272) 103 (41-270)
Norovirus alone	29 (11.5%) 23	223	143 (107-331) 217 (98-430)
Adenovirus alone	19 (7.5%) 14	233	146 (26-335) 248 (35-335)
Negative for all pathogens in the xTAG GPP	69	168	142 (89-369)

**Fig 1:**
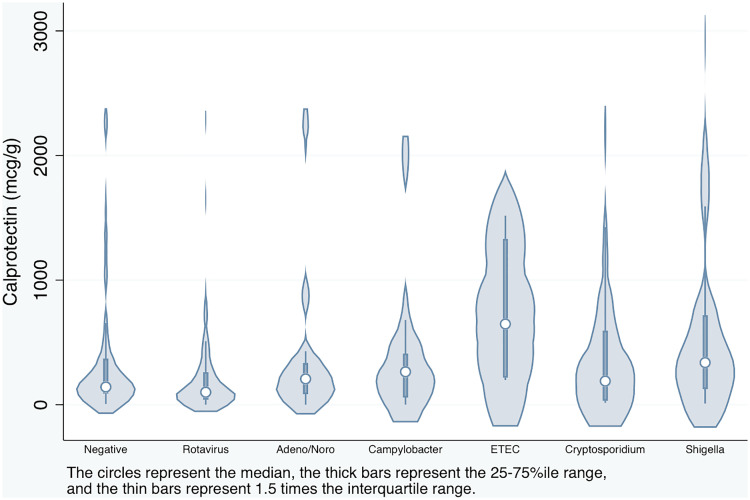
Distribution of fecal calprotectin by enteropathogen. When comparing stools in which different pathogens were detected, fCal levels did not significantly differ except between *Shigella-*positive stools and rotavirus-positive stools (fCal difference 392 μg/g higher, p = 0.004). ‘Alone’ refers to the detection of the pathogen of interest without detection of the other bacterial/parasitic pathogens listed in the table. Pathogens in the xTAG GPP include *Shigella, Campylobacter,* ETEC, *Cryptosporidium, Salmonella,* rotavirus, norovirus, adenovirus 40/41, *E. coli* O157:H7, Shiga toxin-producing *E. coli* (stx1 and stx2 targets), and *Yersinia* (test results for *C. difficile* were not taken into account).

Participants had a median age of 8.7 months (25–75%ile 5.3–13.2 months). There were 35 (11.2%) with severe acute malnutrition. Stools tested had a median fCal of 157 μg/g (25–75%ile 56.9–402.0 μg/g). The fCal distribution was highly skewed to the right, with 10% of samples having fCal > 1033.2 μg/g. There was no significant relationship between fCal and age, either as a continuous variable or a categorical variable. Fig 1 demonstrates the distribution of fCal in samples for each enteropathogen.

## Discussion

In children hospitalized with severe AGE in Botswana, stools with *Shigella* detected had significantly higher fCal than those with rotavirus detected. As these two pathogens are likely the most important causes of AGE in high-burden settings [25], our results are of interest to those seeking to develop care pathways to better identify and treat *Shigella* enteritis and limit associated mortality and morbidity.

Our results add to existing evidence that intestinal inflammation correlated by fCal may be higher in bacterial than viral AGE (6,11–16). Given the propensity for *Shigella* spp. to cause inflammatory colitis, it is not surprising that fCal was higher in stools with *Shigella* detected. ETEC-positive stools had generally higher fCal than *Shigella*-positive stools, but the number of ETEC-positive samples in our study was very small, limiting the precision of these estimates. The median fCal from *Campylobacter* and *Cryptosporidium-*positive stools was also higher than those of virus-positive stools and multiplex-negative stools; however, there was substantial overlap of the distributions with relatively frequent outliers, and no significant differences observed. Therefore, our results are not sufficient to suggest that fCal can reliably identify children with severe AGE that harbor non-*Shigella* bacterial pathogens or *Cryptosporidium*.

This is the largest cohort we are aware of that has compared fCal in children with bacterial and viral enteritis. Prior studies comparing fCal in bacterial and viral AGE were predominantly conducted in higher-resource settings, contextually different from where the majority of the burden of diarrheal disease occurs. There has been one recently-published study enrolling patients in Chandigarh, India, that compared fCal levels in 21 children with bacterial enteritis to 14 children with viral enteritis; however, few conclusions could be drawn due to the small sample size, limited stool microbiologic characterization, and categorization of stools as simply ‘viral’ or ‘bacterial’ [26]. Since the microbiology of acute diarrhoeal disease is fundamentally different in children living in higher-income countries (i.e., the incidence of bacterial or parasitic enteritis is dramatically lower), the role and potential utility of fCal testing is completely different in resource-limited settings. Our results would suggest that there may be a role for the use of fCal testing to guide antimicrobial use in LMICs, where *Shigella* spp. and rotavirus are responsible for a large proportion of clinically significant enteritis. We found the distribution of fCal observed in the samples that were xTAG GPP-negative to be much higher than would be expected for healthy children of a comparable age in higher-income countries [27]. This may be because these stools were positive for an enteropathogen not assayed for (e.g., enteroaggregative *E. coli*) or because of underlying intestinal inflammation related to environmental enteric dysfunction (EED) [28]. The few studies done in lower-resourced settings suggest higher variability and central tendency of fCal among apparently healthy children [29–31]. A Chinese study showed low-income infants in rural areas to have significantly higher fCal levels compared to their affluent urban counterparts and higher fCal levels were negatively associated with growth [31]. A study of low-income Guatemalan children recruited from urban daycares found significantly higher median fCal levels compared to previously described reference levels in the literature in context of frequent *Giardia* detection and linear growth stunting (though there was no relationship between the presence or intensity of *Giardia* infection and fCal level) [29]. Intestinal inflammation may be triggered by a milieu of environmental stimuli, including abnormal intestinal colonization, recurrent parasitosis and dietary factors, rather than solely specific illnesses [29]. Even within the same region, heterogeneity in gut pathogen burden and inflammation has been shown, with marginalized groups more vulnerable to infection and EED [30].

We did not find any association between fCal and age, comparing both age as a binary variable (infant vs. older than 12 months) and age as a continuous variable. This was surprising, given that fCal has been shown to be higher in young infants [27]. However, there were very few infants < 90 days and children > 5 years of age in our analysis, which may have minimized expected differences across age groups. Alternatively, the AGE and/or EED may have elevated fCal to such a degree that expected age-related differences were obscured.

Our study had several limitations. Stool samples were tested with different assays, so some stools underwent more rigorous testing than others, and none of the assays used detected all circulating enteropathogens (e.g., enteroaggregative or enteropathogenic *E. coli*). We could not definitively ascribe causation of the clinical diarrheal disease experienced by participants to any of the pathogens identified, as there was not pre-illness stool testing done or quantitative pathogen ascertainment (since detection of multiple pathogens in children with AGE in high-burden settings are common) [8,21,25]. There are some pathogens (such as *Shigella* and rotavirus) that have been found to be much more likely to be causative of disease when isolated in stool (20); it is therefore not surprising that we did not have any samples in which both *Shigella* and rotavirus were detected. Finally, since fCal is influenced by host and environmental factors, our results may not reflect fCal distributions in different geographical settings.

## Conclusions

In conclusion, among children hospitalized with AGE in Botswana, fCal was significantly higher in those with detectable *Shigella* infection compared to those with detectable rotavirus infection. fCal may be a useful aid to identify children with severe diarrheal disease who could benefit from antibiotic treatment to reduce growth failure and mortality.

## Supporting information

S1 Table Enteropathogen detection and fCal levels, stratified by original prospective study. (DOCX)
