# Supplementary material for: Correlation of fecal calprotectin levels with the detection of treatable enteric pathogens in children with severe acute diarrheal disease in Botswana
Source: PLoS One. 2025 Aug 21;20(8):e0328764. doi: 10.1371/journal.pone.0328764 (PMC12370036; doi:10.1371/journal.pone.0328764)
Supplement: S1 Table — (DOCX) [file pone.0328764.s001.docx]

S1. Supplemental Table.

| Pathogen | # stools positive (study 1) | Fecal calprotectin (μg/g) | # stools positive (study 2) | Fecal calprotectin (μg/g) |
| --- | --- | --- | --- | --- |
| *Shigella*  alone | 22  18 | 338 (105-843)  338 (104-1571) | 12  2 | 360 (145-649)  341 (137-544) |
| *Campylobacter*  alone | 5  0 | 107 (88-313) | 23  7 | 276 (78-580)  253 (38-680) |
| ETEC  alone | 5  0 | 245 (126-449) | 8  2 | 648 (208-1247)  648 (303-992) |
| *Cryptosporidium*  alone | 23  16 | 107 (27-560)  131 (27-568) | 6  2 | 820 (717-1162)  1150 (873-1426) |
| *Salmonella*  alone | 5  4 | 125 (44-143)  85 (23-134) | 2  1 | 91 (74-108)  108 |
| Rotavirus  alone | 100  97 | 102 (39-272)  103 (41-270) | 0 |  |
| Norovirus  alone | 29  23 | 143 (107-331)  217 (98-430) | 0 |  |
| Adenovirus  alone | 19  14 | 146 (26-335)  248 (35-335) | 0 |  |
| Negative for all pathogens in the xTAG GPP | 69 | 142 (89-369) | 0 |  |
